# Rush Medical College and the National Medical Association

**Published:** 1847-12

**Authors:** 


### PART IV.—EDITORIAL.

### ARTICLE I.

### RUSH MEDICAL COLLEGE AND THE NATIONAL MEDICAL ASSOCIATION.

At a meeting of the Faculty of Rush Medical College, on the 3d of January, 1848, the following preamble and resolutions were unanimously adopted.

Whereas, a National Medical Association has been organized for the purpose of advancing the interests of the Profession, whose recommendations have thus far been generally judicious and worthy of adoption; therefore

Resolved, That the Faculty recommend to the board of Trustees the creation of a Chair of Physiology and Pathology and to increase the number of Professors to seven, and that a course of Medical Jurisprudence be added to those now given in this Institution.

Resolved, That attendance upon a Hospital during one session, and the pursuit of disections for twelve weeks shall be required of all candidates for graduation.

Resolved, That we stand ready to comply with the remaining resolutions of the Association, so soon as they shall be generally adopted by the medical schools of the West, or when it shall be apparent that the interests of the Profession require it.

The following persons were then appointed delegates to attend the meeting of the National Association, at Baltimore on the first Monday of May, 1848, ——, ——.

*Remarks.*—In adopting the above resolutions, the Faculty are going but little beyond what they had already done before the organization of the National Association. They had already procured a full and separate course on Physiology. In regard to dissecting, there was little occasion to render it imperative, for so great are the facilities, and such is the attention devoted to this branch, that more than half the class are usually occupied during the lecture term in the dissecting room. At the Hospital, visits are made daily, and the number in attendance is only limited by the difficulty of getting access to the bed side, and the class in attendance is divided into sections, which succeed each other. As, however, this seems not to be the practice elsewhere, we have made obligatory what was before done from choice.

In regard to the recommendation that the student should be required to study three years, lecture terms included, this was already among the requirements of Rush Medical College. So in relation to the adopting of means to ascertain whether students are in attendance upon the lectures; leaving them is so little practiced, that it is not thought necessary at the present time to take any action upon it.

The lengthening the lecture term from four to six months, which is one of the recommendations of the Association, was taken into consideration, but it was regarded as a question upon the propriety of which, doubts might be entertained. The lectures are now given sixteen full weeks without devoting one to introductories at the commencement and another to examinations at the close of the term, and it was thought best to make the increase gradually, by first adding two weeks in order to ascertain whether students would be able to attend longer than sixteen weeks. Action on the question was deferred until correspondence could be had with other medical schools. In the mean time, in order to give such students as are disposed to do so, the opportunity of attending a longer term than the present, a practical course on Clinical Medicine and Surgery, and a course on Anatomy and Physiology during the months of March and April, will be given. If a large proportion of the Winter's class can be induced to attend, it will afford strong encouragement for lengthening the term to six months.

